# Notes and News

**Published:** 1895-03-09

**Authors:** 


### Notes and News.

Collected and Communicated.

### HOSPITAL MEETINGS.

Royal Hospital for Women and Children.—Annual meeting at the Mansion House, on March 15th, at 3.0 p.m.

London Dental Hospital.—Annual meeting at the Hospital, on Wednesday, March 13th, at 6 15 p.m.

Royal Sea Bathing Infirmary.—General Court, on Monday, March 11th, at 3.0 p.m.

Cholera is reported to have broken out at Rosario and Santa Fé, in Argentina. Several deaths are recorded from both places.

A bazaar in aid of the funds of St. Mary's Hospital is to be held in July next. The last bazaar held for the purpose took place twenty-eight years ago, and we trust the one at present proposed will be equally successful.

Lady Hicks and Mrs. Stone—widows of late treasurers of St. Thomas's Hospital—still take a kindly interest in this noble institution, and each has generously contributed £100 to the special fund now being raised for the purpose of re-opening the wards now closed. Mrs. A. T. Hall has contributed £1,000 (in memory of her late husband), and the Canon Brewery Company have contributed £100 for the same purpose.

An eminent psychologist has just passed away in the person of Dr. Hake Tuke, who died in London on the 5th inst. Dr. Tuke, like his grandfather before him, devoted his life to the study of insanity, to the literature of which subject he has added some most valuable contributions. He was editor of the *Journal of Mental Sciences*, and occupied several important posts in England, whilst his labours and researches achieved an European reputation.

For the purpose of considering the question of founding a national institution for the treatment of patients from pulmonary diseases, a meeting has been held at the Langenbeck House, Berlin. The meeting was inaugurated at the instance of the National Association for the Improvement of Public Health, and amongst those who testified by their presence to their sympathy in this movement were the Imperial Chancellor and his son. Professor Leyden, who addressed the Assembly, subsequently appointed a committee for carrying the scheme into effect.

The Board of Management of the Chelsea Hospital for Women have arranged to place a bed in the hospital and one at the Convalescent Home in connection with it at the disposal of the Council of the Newspaper Press Fund for the use of the mothers, wives, and daughters of distressed members of the journalistic profession.

We regret to record the death of the eminent surgeon, Sir William Savory, who died at his residence in Brook Street on the 4th inst. Born in 1826, he took his M.B. degree at the London University at the age of twenty-two, and commenced a brilliant career, in the course of which he became Fellow of the Royal College of Surgeons, Professor of Comparative Anatomy and Physiology to the same; Fellow of the Royal Society; President of the Royal College of Surgeons; Surgeon-extraordinary to the Queen; and was created a baronet in 1890. Besides possessing these marks of distinction, he held important posts in surgery, acting as surgeon or consulting-surgeon at St. Bartholomew's, the Great Northern, and the London fever hospitals. Sir William Savory contributed some useful works to medical literature, he was a man of action rather than of theories. His progress through his wards was marked by a rapidity which demanded a quick intelligence on the part of his class. Keen and incisive in judgment, he inspired confidence in those under him, a confidence which was heightened by his immense practical experience and commanding appearance. Sir William Savory had retired from some of his most arduous appointments before his death, although he was engaged in active practice until overtaken by illness.

The Pathological Society devoted itself on Tuesday evening to the consideration of diphtheria. Dr. Bertram Hunt read a careful paper on the pathology of the disease, entering more especially into the question of the mode by which immunity was attained and the nature and *modus operandi* of the immunising substance. Contrary to what had been held by some, the drift of his argument went to show that this material, the so-called antitoxin, was not a direct product of the animal cells, but was in some form a derivative of the action of the cells upon the toxin which had been introduced, a beneficent modification of a toxin. Animals which were naturally immune to any particular disease were entirely indifferent to the toxins produced by the microbes which were their cause, and thus produced no antitoxin. Animals, on the other hand, which were susceptible, might, if their susceptibility were extreme, at once succumb, but if they did not they were protected by the production of an antitoxic substance which was special and specific for each disease, and was really a product or modification of the toxin itself, a substance which was chemically useful to the cells of the organism in resisting the bacteria and their products. The greater the effort or struggle of the animal to resist the toxin, the greater the amount of the antitoxic material which was produced. The action of antitoxin was not as a chemical antidote. Although it was true that in some cases a mixture of antitoxins with toxins in a test tube might prevent the action of the latter, this was not the result of a chemical neutralisation. The two substances existed side by side, and the want of effect when they were injected simultaneously was the result of opposed and balanced actions on the organism. The paper was listened to with great interest by a large assemblage of members.

